# Malignant catatonia due to anti-NMDA-receptor encephalitis in a 17-year-old girl: case report

**DOI:** 10.1186/1753-2000-5-15

**Published:** 2011-05-13

**Authors:** Angèle Consoli, Karine Ronen, Isabelle An-Gourfinkel, Martine Barbeau, Donata Marra, Nathalie Costedoat-Chalumeau, Delphine Montefiore, Philippe Maksud, Olivier Bonnot, Adrien Didelot, Zahir Amoura, Marie Vidailhet, David Cohen

**Affiliations:** 1 Department of Child and Adolescent Psychiatry, Université Pierre et Marie Curie, Hôpital Pitié-Salpêtrière, AP-HP, 47-83, boulevard de l'Hôpital, 75013, Paris, France; 2 Department of Neurology, Université Pierre et Marie Curie, Hôpital Pitié-Salpêtrière, AP-HP, 47-83, boulevard de l'Hôpital, 75013, Paris, France; 3 Department of Internal Medicine, Université Pierre et Marie Curie, Hôpital Pitié-Salpêtrière, AP-HP, 47-83, boulevard de l'Hôpital, 75013, Paris, France; 4 Department of Nuclear Medicine, Université Pierre et Marie Curie, Hôpital Pitié-Salpêtrière, AP-HP, 47-83, boulevard de l'Hôpital, 75013, Paris, France; 5 Department of Adult Psychiatry, Université Pierre et Marie Curie, Hôpital Pitié-Salpêtrière, AP-HP, 47-83, boulevard de l'Hôpital, 75013, Paris, France; 6 Reference center of paraneoplastic neurological syndrome diagnosis and treatment, Hôpital Pierre Wertheimer, 59, bld Pinel, 69 003 Lyon

**Keywords:** Anti-NMDA-Receptor encephalitis, Adolescence, Malignant catatonia

## Abstract

Anti-NMDA-Receptor encephalitis is a severe form of encephalitis that was recently identified in the context of acute neuropsychiatric presentation. Here, we describe the case of a 17-year-old girl referred for an acute mania with psychotic features and a clinical picture deteriorated to a catatonic state. Positive diagnosis of anti-NMDA-receptor encephalitis suggested specific treatment. She improved after plasma exchange and immunosuppressive therapy. Post-cognitive sequelae (memory impairment) disappeared within 2-year follow-up and intensive cognitive rehabilitation.

## Background

NMDA receptors are ligand-gated cation channels that play an important role in synaptic plasticity [1] and seem to be implicated in the physiopathology of neuropsychiatric disorders [2]. NMDA receptors are heteromers of NR1 and NR2 subunits (A, B, C or D) that bind glycine and glutamate, respectively [3]. Both glycine and glutamate must bind for the NMDA receptor to be functional. Anti-NMDA-receptor encephalitis has been recently identified. The antibodies found in anti-NMDA-receptor encephalitis are directed against the NR1 subunit of the NMDA receptor [4].

The clinical syndrome of a paraneoplastic neuropsychiatric disorder associated with ovarian teratoma was first described in 2005 [5], and Dalmau and colleagues identified and described the specific antibody in 2007 [6]. Since then, several case reports of anti-NMDA-receptor encephalitis have been published, suggesting that this illness is not rare [4,7-11]. In 2008, Dalmau and al. published a series of 100 cases of anti-NMDA-Receptor encephalitis [12]. Recently, the same group reported on more than 400 patients with anti-NMDA-Receptor encephalitis over a 3-year period [4]. The exact incidence of anti-NMDAR encephalitis is unknown, but it seems to be more frequent than any other known paraneoplastic encephalitis [4]. It predominantly affects children and young adults and may occur with or without tumor association [4]. Eighty percent of the patients are women. The clinical syndrome is now clearly described. First, a brief viral-like episode (e.g., headache, hyperthermia) can occur. This is followed by an acute phase that includes neuropsychiatric symptoms such as agitation, psychotic symptoms (i.e., delusions or hallucinations), behavioral changes, generalized or partial seizures, progressive unresponsiveness, abnormal movements (e.g., dyskinesia), dysautonomy and hypoventilation that can require ventilation assistance and intensive care. The frequency of tumors varies according to age, sex and ethnicity [4]. Usually teratoma of the ovaries in women or testicular tumors in men that express NMDA-R which triggers antibody production, are found [13].

For patients with anti-NMDA-Receptor encephalitis, magnetic resonance imaging (MRI) scans are often normal or show only minor, non-specific signs. Patients' cerebrospinal fluid (CSF) may show pleocytosis and an elevated protein concentration. In addition, patients' electroencephalogram (EEG) results exhibit diffuse slow activity. Despite a severe initial presentation, complete or near complete recovery can be reached using immunosuppressive therapy and tumor resection; however, severe sequelae and even death occur in up to 25% of all cases [12].

In this paper, we present a case report of a 17-year-old girl referred for acute mania with psychotic features and malignant catatonia due to anti-NMDA-Receptor encephalitis. She was first treated empirically with immunosuppressive therapy and plasma exchange (PE) for presumed immune mediated encephalitis based on increased antinuclear antibodies. Treatment was then continued based on the diagnosis of anti-NMDA-R encephalitis.

## Case Presentation

A 17-year-old girl with no medical, psychiatric or surgical history began exhibiting symptoms of hypochondriasis. Her parents reported that she had sudden changes of mood, becoming more irritable and sensitive. In a few days, she began to get worse. She presented manic symptoms with psychomotor excitement, logorrhea, tachypsychia, euphoric state and insomnia. She had delusions and hallucinations with dysmorphophobic and nosophobic thematics. She also presented with one generalized seizure, although she did not suffer from epilepsy.

**Figure 1:**
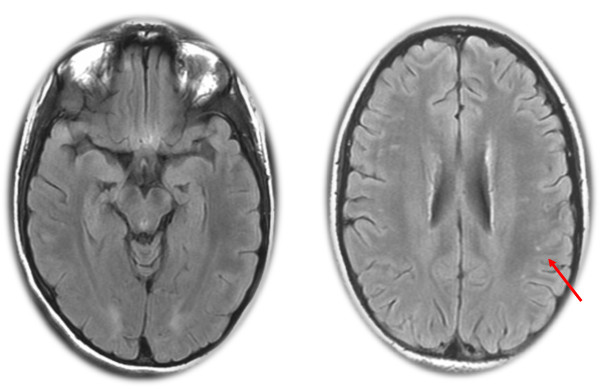
**Small and non-specific hyperintensities in Magnetic Resonance Imaging (MRI)**.

**Figure 2:**
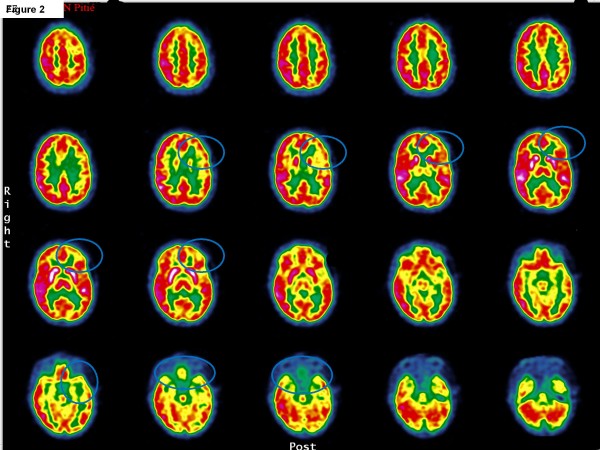
**Left frontal-temporal cortex hypometabolism and moderate bilateral hippocampic hypometabolism in cerebral positron emission tomography (^18^FDG-PET)**.

The patient was transferred to the closest psychiatric department where she presented with catatonia syndrome without extrapyramidal signs. She was given olanzapine (40 mg/day), loxapine (50 mg/day) and clonazepam (3.5 mg/day). She soon showed malignant catatonia with autonomic instability, fever, arterial hypertension and CPK increase (4500 UI/L) and was transferred to the university department of adolescent psychiatry. Antipsychotic medications were stopped, and a high dosage of lorazepam (15 mg/day) was started. Because of her life-threatening condition, the patient was transferred to an intensive care unit. Dysautonomy and fever improved, but she remained catatonic, showing rigidity, mutism, staring, waxy flexibility and negativism. An exhaustive biological check-up was conducted to rule out possible organic causes (i.e., immunological, infectious, metabolic, iatrogenic and toxic) [14]. An examination of her cerebral spinal fluid revealed eight cells, and an electroencephalogram showed diffuse slow waves (0.5 to 1 wave per second); antinuclear factors were positive (1/320), but anti-DNA antibodies were not. A Magnetic Resonance Imaging (MRI) scan showed subtle, small and non-specific hyperintensities (Figure 1). A cerebral positron emission tomography (^18^FDG-PET) revealed left frontal-temporal cortex hypometabolism and moderate bilateral hippocampic hypometabolism (Figure 2). Electroconvulsive therapy (ECT) was postponed due to arguments supporting hypothesis of acute encephalitis (seizures, EEG signs and brain hypometabolism). Based on suspicion of neuropsychiatric systemic lupus erythematosus (SLE) (because of positive antinuclear factors and neurological symptoms), immuno-suppressive therapy was initiated. For 3 days, she received prednisone at a dose of 1 g IV. This was followed by a month of 1 mg/kg/day oral prednisone, which was progressively decreased. Two weekly pulses of cyclophosphamide (0.7 g/m^2^) and 13 plasma exchanges were also given. Antiepileptic treatment was added to the immunosuppressive treatment given the recent general seizures in the context of encephalitis.

Catatonia as well as affective and psychotic symptoms progressively improved, but the patient revealed many neurological sequelae. Indeed, she presented with frontal lobe syndrome (perseverations, grasping, lack of emotions, lack of initiative, speech reduction and aphasia), severe impairment of memory, ataxia, stereotyped movements (e.g., chewing movements and teeth grinding), right ptosis and myosis. Early neuropsychological and speech testing confirmed the presence of frontal-like aphasia with perseverations, significant slowness, severe verbal and non-verbal impairment, major memory impairment (in short term and working memory) with confabulations. Furthermore, she was anosognosic. Her cognitive functions were clearly abnormal given her age and school level before this episode (Table 1).

**Table 1:** Course of cognitive assessments after plasma exchange and immunosuppressive treatment

Time	PE ending*	6 months	10 months	14 months
Prednisone (mg per day)	1	0	0	0

Cyclophosphamide (0.7/m^2^)	2	0	0	0

Plasma exchanges (N received previously)	13	0	0	0

Immunoglobulin (N of cure)	0	3	3	0

**WAIS III ***Verbal comprehension index*				
Similarities	14	17	17	
Arithmetic	Impossible	8	8	
Vocabulary	7	15	15	
Information	8	8	8	13
Comprehension	12	16	16	
*Working memory index*				
digit span	1	7	7	
**Verbal IQ**	92	111	111	
*Perceptual organization index*				
Matrix reasoning	6	7	11	13
Picture completion Block design matrix	6 1	10 8	10 8	
*Processing speed index*				
Symbol search	3	11	11	
Digit symbol-coding	10	Refusal	10	
**Performance IQ**	63	100	109	
**Total IQ**	Not valid	107	111	
**Wechsler Memory (immediate/delayed recall)**				
Logical memory	2/1		2/6	10/8
Face recognition	2/13		2/13	
Verbal paired associates	3/1		3/12	7/12
Family pictures	6/1		6/1	
Letter-number sequencing	1		1	7
Spatial span	1		1	

PE = Plasma exchange; IQ = Intellectual Quotient; WAIS = Wechsler Adult Intelligence Scale

After the neurological sequelae indicated an encephalopathy with frontal and limbic dysfunction, we evaluated her serum and cerebrospinal fluid for an increase in anti-NMDA-Receptor antibodies. These levels were highly elevated. The anti-NMDA-Receptor encephalitis diagnosis was therefore retained, yet no tumor was found. The patient was transferred to a recovery center, where she received speech therapy and cognitive remediation for her memory loss. Immunosuppressive treatment with intravenous immunoglobulin was prescribed for the next months (Table 1), and she continued to improve. Post-cognitive sequelae disappeared within 2 years. Subsequently, she was able to return to school and seemed to have regained her original cognitive abilities (Table 1).

## Conclusions

In this case report, acute mania with psychotic features deteriorating to catatonic state revealed anti-NMDA-receptor encephalitis. This encephalitis is a multistage illness that progresses from psychosis, memory deficits, seizures, and language disintegration to a state of unresponsiveness with catatonic features [4]. Psychiatric symptoms, including delusions, anxiety, insomnia, and mania, can occur initially, and they usually present less than 2 weeks after prodromal symptoms (headache, fever, nausea, diarrhea or upper respiratory tract symptoms) [4]. The patients are often initially seen by psychiatrists. Anti-NMDA-Receptor encephalitis predominantly affects children and young adults [4,13] and may or may not be associated with a tumor. Approximately 80% of patients are women. The presence of a tumor is more frequent in women who are older than 18 years and who are black [4]. Because of the initial psychiatric presentation, high dosages of antipsychotics were administrated to the patient. Even if the role of antipsychotics is not clear and that catatonic features occur in anti-NMDA-Receptor encephalitis, they may aggravate the catatonic symptoms.

Therapeutic approaches to catatonia are mainly symptomatic. It is recommended to use high dosages of benzodiazepines and to perform electroconvulsive therapy in case of resistance or a life threatening condition [15]. Treatment of the causal organic condition is also warranted. In this case, the patient did not respond to high doses of lorazepam. ECT was discussed but postponed due to signs of acute encephalitis.

A recent literature review showed that organic causes of catatonia in young people make up 20% of all cases [14], and this rate was confirmed in a prospective study [16]. Among the organic causes, encephalitis, caused by infection or immune mechanisms requires specific treatments. In particular, neuropsychiatric systemic lupus erythematosus (SLE) can be revealed by a catatonic syndrome. It is crucial to diagnose and begin an appropriate treatment quickly to improve the patient's prognosis. Plasma exchange appears to be an efficient treatment option in SLE and catatonia, and it avoids the use of ECT [17,18].

In the present case, given the increased antinuclear antibodies, the MRI results (non-specific white matter hyperintensities) and the PET hypometabolism, clinicians first hypothesized the presence of SLE. The presence of antinuclear and/or thyroid peroxidase antibodies, in addition to NMDAR antibodies, has been previously described, most typically in children and can suggest a predisposition to this type of auto immunity [4,13]. Associated plasma exchanges and immunosuppressive therapy were used as treatment. After improvement of the malignant catatonia, neurological markers led to a diagnosis of anti-NMAD-receptor encephalitis, which indicated the need for continuation of immunomodulatory therapy with immunoglobulins. This treatment led to a major improvement in catatonic, psychiatric and neurological symptoms. Management of anti-NMDAR encephalitis is focused on immunotherapy and the detection and removal of a teratoma [4]. Based on an extensive review (400 patients over a 3-year period), Dalmau and colleagues proposed an algorithmic strategy to guide treatment [4]. The first line of immunotherapy consists of corticosteroids, intravenous immunoglobulins, and plasma exchange (alone or in combination). The second line of immunotherapy (rituximab or cyclophosphamide or both) is usually needed in the case of a delayed diagnosis or in the absence of a tumor [4].

Regarding the patient's cognitive impairment, NMDA receptors are known to play a crucial role in synaptic plasticity, which is involved in memory, learning and cognition [19]. Disruption of these receptors can result in seizures and changes in memory, learning and behavior [1]. It is possible to explain the patient's sequelae by a diagnosis of diffuse encephalopathy that is mainly due to frontal and limbic dysfunction. Similarly, an early manifestation with acute severe psychiatric signs and catatonia may be related to dysfunction of the NMDA-R circuitry, as the NMDA-R has been implicated in psychotic symptomatology [20,21].

Interestingly, another case of adolescent catatonia associated with encephalitis has been reported [22]. In that case, encephalitis was paraneoplastic and improved after an ovarian tumor ablation. In retrospect, it is possible that that case also presented was anti-NMDA-receptor encephalitis, given its frequent association with ovarian teratoma [12,13]. Another recent case report showed an excellent recovery after immunotherapy (plasma exchange and corticosteroids) in a case of anti-NMDAR encephalitis in a 12-year-old girl [23].

Recognition of encephalitis by psychiatrists is important because patients may initially present with psychiatric symptoms and catatonic features. Here, symptoms and paraclinical data are in accordance with cases of anti NMDAR encephalitis already reported: severe psychiatric symptoms, seizures, orofacial dyskinesia. In the case of severe and possible life-threatening anti-NMDA-receptor encephalitis, it is essential that a quick and adapted treatment is implemented. Indeed, the prognosis of anti NMDA-R encephalitis varies: 75% of cases recover with immunotherapy and tumor ablation (when present), while 25% of cases lead to severe sequelae and even death [4]. Relapse occurs in 15% of all cases [12].

This case report emphasizes the importance to search for a medical condition in catatonic syndrome of young people to treat and avoid severe neurological sequelae or death. The proposal of the DSM-V workgroup to make catatonia a "specifier" added as a fifth digit to other diagnoses seems likely to reduce rather than enhance clinician awareness of importance of recognizing this syndrome and researching for medical condition, particularly during psychiatric training. By contrast, a large group of experts advocated a unique and broadly-defined code for catatonia as a syndrome, which can be diagnosed acutely in addition to any suspected or established associated disorders [24]. In this case, the initial psychiatric clinical presentation was complicated by a malignant catatonic state, which is now well-described in anti-NMDA-Receptor encephalitis. Child psychiatrists need to know that anti-NMDA-Receptor encephalitis occurs frequently in children and adolescents. Plasma exchanges and immunosuppressive therapy treatments can dramatically improve catatonic syndrome associated with autoimmune dysfunction such as SLE [17,18], PANDAS [25] and NMDA-receptor encephalitis.

## Competing interests

The authors declare that they have no competing interests.

## Authors' contributions

AC, KR and DC drafted the manuscript. AC, KR, IA, DM, NC, DM, OB, ZA, MA and D participated in collecting and discussing clinical data. MB carried out cognitive assessment and discussion. AD, PM performed imagery, laboratory investigations and discussed them. All authors read and approved the final manuscript

## Consent statement

Written informed consent was obtained from the patient for publication of this case report and accompanying images. A copy of the written consent is available for review by the Editor-in-Chief of this journal.
